# 

**Published:** 1882-05

**Authors:** 


### Pamphlets.

The Use of Constitutional Remedies in the Treatment of Ear Diseases. By Samuel Theobald, M.D. Reprint from *The Medical News*.

Anæsthesia and Non-Anæsthesia in the Extraction of Cataract; with some practical suggestions regarding the performance of the operation, and comparative statistics of two hundred cases. By Haskel Derby, M.D., Cambridge: Riverside Press. 1882.

Gunshot Wound of Abdomen; Fecal Fistula—Spontaneous Closure. By A. Sibley Campbell, Augusta. Reprint from Trans. Med. Asso. of Georgia.

Proceedings of the Medical Society of the County of Kings, N. Y.

A New System of Surgical Mechanics. By Charles F. Stillman, M.D., New York. Reprint from Transactions of American Medical Association, 1881.

On Ovariotomy. By Thos. Keith, Esq., M.D., F.R.C.S.E.

Dr. Richard O. Cowling. Valedictory Address at the Commencement of the University of Louisville. By D. W. Yandell, M.D.

Epidemic Convulsions. By D. W. Yandell, M.D.

Intermittent Spinal Paralysis of Malarial Origin. By V. P. Gibney, M.D., New York.

A New Idea in the Treatment of Fractures of the Femur. By Charles H. Harris, M D., Cedartown, Ga.

